# Nonlinear optical properties of arsenic telluride and its use in ultrafast fiber lasers

**DOI:** 10.1038/s41598-020-72265-3

**Published:** 2020-09-17

**Authors:** Jinho Lee, Young In Jhon, Kyungtaek Lee, Young Min Jhon, Ju Han Lee

**Affiliations:** 1 School of Electrical and Computer Engineering, University of Seoul, Seoul, 02504 South Korea; 2 Sensor System Research Center, Korea Institute of Science and Technology, Seoul, 02792 South Korea

**Keywords:** Nonlinear optics, Nanoparticles, Fibre lasers, Mode-locked lasers

## Abstract

We report the first investigation results of the nonlinear optical properties of As_2_Te_3_. More specifically, the nonlinear optical absorption properties of the prepared α-As_2_Te_3_ were investigated at wavelengths of 1.56 and 1.9 μm using the open-aperture (OA) Z-scan technique. Using the OA Z-scan technique, the nonlinear absorption coefficients (β) of α-As_2_Te_3_ were estimated in a range from (− 54.8 ± 3.4) × 10^4^ cm/GW to (− 4.9 ± 0.4) × 10^4^ cm/GW depending on the irradiance of the input beam at 1.56 μm, whereas the values did from (− 19.8 ± 0.8) × 10^4^ cm/GW to (− 3.2 ± 0.1) × 10^4^ cm/GW at 1.9 μm. In particular, the β value at 1.56 μm is an order of magnitude larger than the previously reported values of other group-15 sesquichalcogenides such as Bi_2_Se_3,_ Bi_2_Te_3,_ and Bi_2_TeSe_2_. Furthermore, this is the first time report on β value of a group-15 sesquichalcogenide at a 1.9-μm wavelength. The density functional theory (DFT) calculations of the electronic band structures of α-As_2_Te_3_ were also conducted to obtain a better understanding of their energy band structure. The DFT calculations indicated that α-As_2_Te_3_ possess sufficient optical absorption in a wide wavelength region, including 1.5 μm, 1.9 μm, and beyond (up to 3.7 μm). Using both the measured nonlinear absorption coefficients and the theoretically obtained refractive indices from the DFT calculations, the imaginary parts of the third-order optical susceptibilities (Im χ^(3)^) of As_2_Te_3_ were estimated and they were found to vary from (− 39 ± 2.4) × 10^–19^ m^2^/V^2^ to (− 3.5 ± 0.3) × 10^–19^ m^2^/V^2^ at 1.56 μm and (− 16.5 ± 0.7) × 10^–19^ m^2^/V^2^ to (− 2.7 ± 0.1) × 10^–19^ m^2^/V^2^ at 1.9 μm, respectively, depending on the irradiance of the input beam. Finally, the feasibility of using α-As_2_Te_3_ for SAs was investigated, and the prepared SAs were thus tested by incorporating them into an erbium (Er)-doped fiber cavity and a thulium–holmium (Tm–Ho) co-doped fiber cavity for both 1.5 and 1.9 μm operation.

## Introduction

Nonlinear optical responses of materials have always been of high technical interest in the area of optics and photonics due to their usefulness in various applications such as optical switching^1^, wavelength conversion^2^, second harmonic generation^3^, and sautrable absorption^4^. The commonly investigated nonlinear optical responses of materials include χ^(2)^ effects^5^, χ^(3)^ effects (also called Kerr effects)^6^, and nonlinear absorption effects^7–9^. Among the aforementioned nonlinear optical properties, nonlinear absorption effects are technical interesting since they can be applied to implementing absorption-related functional devices such as saturable absorbers (SAs)^4^ and multi-photon absorption devices^10^.

Recently, a huge number of investigations have been conducted regarding saturable absorption properties of various emerging materials, more specifically nano-materials. It is well-known that nonlinear saturable absorption effect usually occurs due to the Pauli’s blocking principle within semiconducting materials^11^. Note that the continued rapid growth in pulsed laser-related industries such as material processing, medicine, gas sensing, LIDAR, and free-space communication^12–15^ demands the development of high performance pulsed lasers, which can be realized only if an efficient SA should be used. The ever-increasing technical demand for highly efficient, cost-effective SAs has generated enormous interest in searching for new and novel nonlinear absorption materials.

Until now, most of commercially available SAs have been implemented using III–V compound semiconductors^16^ due to their proven performance and reliability, even if they have the fundamental limitations of limited operating bandwidth and the need for sophisticated/expensive facilities. A large number of investigations into alternative nonlinear optical materials that could overcome these limitations have been conducted so far. A variety of materials, which are mostly nano-structured materials, have been identified as saturable absorption materials suitable for SA implementation. These include: carbon nanotubes (CNTs)^4,17–20^, graphene^21–26^, graphene oxide (GO)^27,28^, topological insulators (TIs)^29–38^, topological semimetal^39^, transition metal dichalcogenides (TMDCs)^40–53^, transition metal monochalcogenides (TMMCs)^54^, filled skutterudites (FSs)^55^, black phosphorus (BPs)^56–58^, gold nano-particles^59–61^, and MXenes^62,63^.

Our research group has investigated the nonlinear saturable absorption properties of a range of materials such as TIs, MXene, and TMDCs. In particular, we conducted a series of investigations into the ultimate potential of various TIs such as Bi_2_Te_3_, Bi_2_Se_3_, and CoSb_3_ as saturable absorption materials. We showed that TIs possess good nonlinear saturable absorption properties whether the materials are nano-structured or bulk-structured.

As an ongoing study, our group has focused on arsenic telluride (As_2_Te_3_), which is another bulk form of group-15 sesquichalcogenides with a generic formula A_2_B_3_ (A = As, Sb, Bi; B = S, Se, Te). As_2_Te_3_ is known to have lattice constants similar to Bi_2_Se_3_. As_2_Te_3_ exists in two crystallographic forms: α- and β-As_2_Te_3_. α-As_2_Te_3_ at ambient pressure is known to have a monoclinic structure with a C2/m space group^64,65^, and it exhibits lower thermoelectric figure of merit (ZT) than Sb- and Bi-based tellurides^66^. On the other hand, β-As_2_Te_3_ with a rhombohedral *R3m* symmetry, which is known to have outstanding thermoelectric properties^67,68^, has been reported to have topologically protected surface states as a three-dimensional TI when the uniaxial strain is along the c-axis of the rhombohedral crystal structure^69,70^.

A number of investigations into the physical properties of group-15 sesquichalcogenides have been conducted in terms of thermoelectric, electronic, vibrational, and optical properties following the recent technical interest in those materials. However, only a few investigations on As_2_Te_3_ have been conducted so far. Those limited investigations were mostly focused on thermoelectric, vibrational, and electronic properties^71–75^ and thus there has been no research on the optical properties of As_2_Te_3_, to the best of our knowledge.

In this work, the nonlinear optical absorption properties of α-As_2_Te_3_ were investigated in both theoretical and experimental ways. First, a series of measurements including scanning electron microscopy (SEM), energy dispersive spectroscopy (EDS), Raman spectrum, and X-ray photoelectron spectroscopy (XPS), were carried out for As_2_Te_3_ particles, to determine the material properties. Second, the electronic and optical properties such as energy band structure, absorption spectrum, and wavelength dependent refractive index values were theoretically calculated with the density functional theory (DFT) calculation method. It is shown that α-As_2_Te_3_ is a semiconducting material with a wide linear absorption bandwidth that can cover the 2-μm wavelength region. Third, the nonlinear absorption coefficients (β) of α-As_2_Te_3_ were measured using the open aperture (OA) Z-scan measurement method at wavelengths of 1,560 nm and 1,900 nm. Subsequently, the imaginary parts of the third-order optical susceptibilities (Im χ^(3)^) of As_2_Te_3_ were estimated at 1,560 and 1,900 nm, using both the measured nonlinear absorption coefficients and the theoretically obtained refractive indices from the DFT calculations. The nonlinear absorption coefficients (β) of α-As_2_Te_3_ varied in a range from (− 54.8±3.4) ×10^4^ cm/GW to (− 4.9±0.4) ×10^4^ cm/GW depending on the irradiance of the input beam at 1,560 nm, whereas the value did from (− 19.8±0.8) ×10^4^ cm/GW to (− 3.2±0.1) ×10^4^ cm/GW at 1,900 nm. The estimated imaginary parts of the third-order optical susceptibilities (Im χ^(3)^) of As_2_Te_3_ were found to vary from (− 39±2.4) ×10^−19^ m^2^/V^2^ to (− 3.5±0.3) ×10^−19^ m^2^/V^2^ at 1,560 nm and (− 16.5±0.7) ×10^−19^ m^2^/V^2^ to (− 2.8±0.1) ×10^−19^ m^2^/V^2^ at 1,900 nm, respectively, depending on the irradiance of the input beam. Fourth, the feasibility of using As_2_Te_3_ as a base material for implementation of a broadband SA was investigated by fabricating an SA on a fiber ferrule-based sandwich structure platform. Using the prepared α-As_2_Te_3_-based SA within an erbium (Er) fiber-based ring cavity, stable mode-locked pulses with a temporal width of ~ 858 fs were generated at a wavelength of ~ 1,559.8 nm. Additionally, mode-locked pulses with a temporal width of ~ 1.34 ps were readily obtained at a wavelength of 1,911.4 nm by using another As_2_Te_3_-based SA within a thulium–holmium (Tm–Ho) co-doped fiber laser ring cavity.

## Results

### Experimental and theoretical investigation of material properties

**Figure 1:**
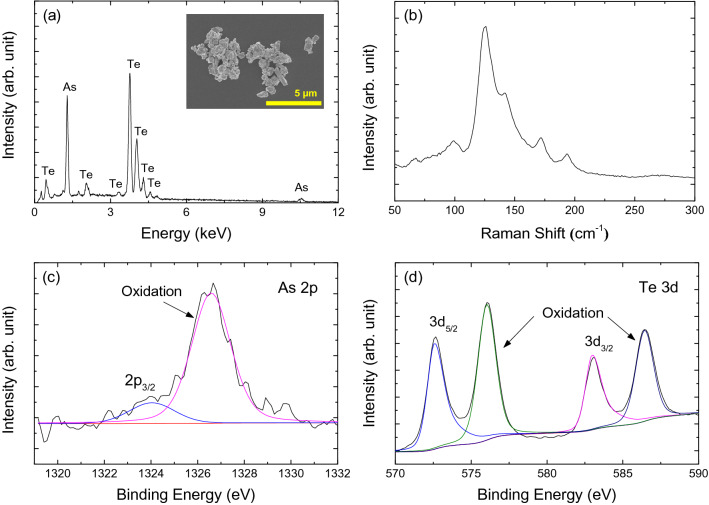
(**a**) EDS spectrum of the As_2_Te_3_ particles. Inset: SEM image of the As_2_Te_3_ particles. (**b**) Raman spectrum of the As_2_Te_3_ particles and XPS spectra of the (**c**) As 2p core level, and (**d**) Se 3d core level.

Commercially available As_2_Te_3_ crystals (LTS Research Lab., 99.99%) were used as a starting material in the present experiment. A bath type ultrasonicator was used to synthesize the α-As_2_Te_3_ particles. The measured energy dispersive spectroscopy (EDS) spectrum of the α-As_2_Te_3_ particles is shown in Fig. 1a. A small amount of α-As_2_Te_3_ particle solution was dropped on top of a slide glass and dried for 24 h for the EDS measurement. The spectrum shows strong peaks corresponding to As and Te. A scanning electron microscope (SEM) image of the α-As_2_Te_3_ particles dried on a silicon substrate is shown in the inset of Fig. 1a. The size of the α-As_2_Te_3_ particles ranged from tens of nanometers to about few micrometers. Figure 1b shows the measured Raman spectrum of the prepared α-As_2_Te_3_ particles. The A^1^ mode E″ mode of Te are shown at 125 cm^−1^ and 141 cm^−1^, and two high-frequency modes were observable at 171 cm^−1^ and 200 cm^−1^, respectively^75^. XPS measurements were conducted to analyze the stoichiometry of the α-As_2_Te_3_ particles. Figure 1c shows the As 2p spectrum, whereas the Te 3d spectrum is shown in Fig. 1d. The peak at ~1,323.9 eV in the As 2p region of Fig. 1c is consistent with the reported binding energy value of the As 2p_3/2_^76^, while the two peak at ~ 572.5 and ~ 583 eV in the Te 3d region of Fig. 1d are consistent with those of Te 3d_5/2_ and Te 3d_3/2_^78^. An additional peak was located at ~ 1326.5 eV in the As 2p region, whereas they were at ~ 576 and 586.4 eV in the Te 3d region. The existence of those additional peaks can be attributed to the oxidation of As and Te atoms on the surface^76–78^.

**Figure 2:**
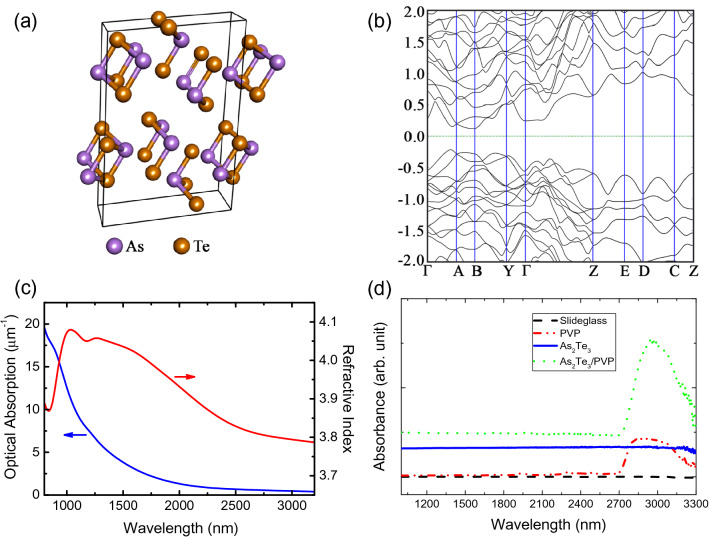
(**a**) Structure of the α-As_2_Te_3_ crystal. (**b**) The calculated electronic band structure of α-As_2_Te_3_. (**c**) The calculated optical absorption and refractive index spectra of α-As_2_Te_3_. (**d**) Measured linear absorption spectrum of α-As_2_Te_3_/PVP composite.

For a deeper investigation of the electrical and optical properties of α-As_2_Te_3_, we performed density function theory (DFT) calculations of the electronic band structures as well as optical refractive and/or absorption spectra of the α-As_2_Te_3_ crystal. The optimized geometry of α-As_2_Te_3_ is shown in Fig. 2a, which belongs to space group C2/m. The calculation showed that the electronic band gap of α-As_2_Te_3_ is around 0.34 eV, indicating a good agreement with previous ab-initio calculations (0.32 eV)^65^ and readily allowing 1,550–1,900-nm saturable absorption (Fig. 2b). To make this point clear, we further investigated the optical refractive and/or absorption properties of α-As_2_Te_3_ using the Kubo–Greenwood formula and were able to obtain consistent results (Fig. 2c). Meanwhile, the previous experiment showed that the bandgap should be 0.46 eV^79^, while the estimation based on temperature-dependent thermoelectric activation underestimated the bandgap to be around 0.13 eV^80^. Considering our calculation results, α-As_2_Te_3_ might be a good SA even for applications requiring longer wavelengths than that of 0.34 eV due to exciton formation, edge-states, and structural defects. In order to check the linear optical absorption of the As_2_Te_3_, the linear absorption measurement was conducted for the α-As_2_Te_3_/polyvinylpyrrolidone(PVP) composite deposited onto a glass slide, and the linear absorption of the α-As_2_Te_3_/PVP composite was measured using a spectrophotometer (UV-3600PLUS, Shimadzu). Figure 2d clearly shows broadband absorption of the α-As_2_Te_3_/PVP composite over a wide spectral range from 1,000 to 3,300 nm. The pure PVP film has a small absorption over a range of 1,000 nm–2,700 nm with two small peaks at 1946 nm and 2,280 nm, respectively, which are caused by the PVP vibration^81^. The interesting fact is that there is a strong peak at ~ 2,950 nm (wavenumber =  ~ 3,400 cm^−1^) in the absorption spectrum of the α-As_2_Te_3_/PVP film, which corresponds to the O–H stretching vibration of PVP^82,83^. Since the uniformity of our prepared sample is not good, it is thus very difficult to figure out the precise thickness value of our sample at a particular position, at which the incident laser beam is focused. Therefore, it is very hard to obtain a precise linear absorption coefficient of our sample. In general, the linear absorption increases as the sample thickness is enlarged, according to Beer–Lambert law (i.e., $T = \sim \exp ( - \alpha \times d)$ where *T* is the transmittance, *α* is the absorption coefficient, and *d* is the sample thickness). The sample-thickness-dependent property variations have been reported for various saturable absorption materials such as TIs, TMDCs, and BP^84–89^. The bandgap energies of the materials are known to change depending on the layer number. Considering the fact that a material with not less than six layers can be regarded as bulk, our used As_2_Te_3_ sample is bulk-structured^87^ since the minimum size of the As_2_Te_3_ particles used in this investigation is tens of nanometers. This indicates that the non-uniformity of our As_2_Te_3_ particles could not induce any associated bandgap change due to its bulk nature.

**Figure 3:**
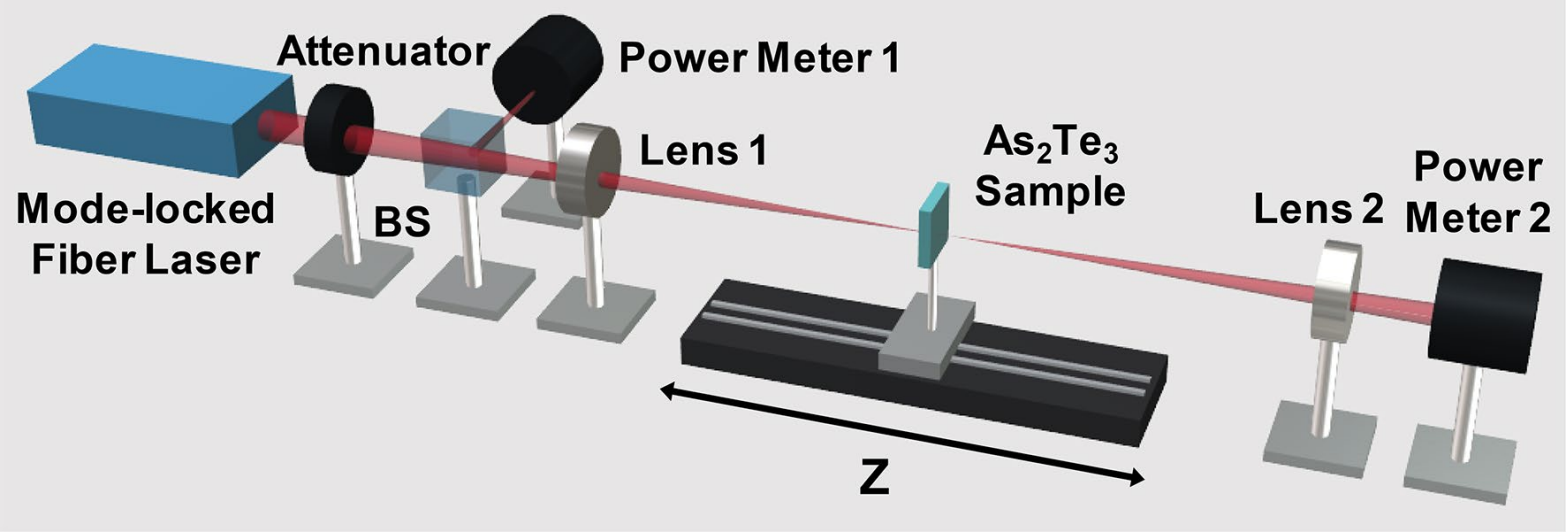
Schematic diagram of the Z-scan experimental setup. *BS*: Beam splitter.

**Figure 4:**
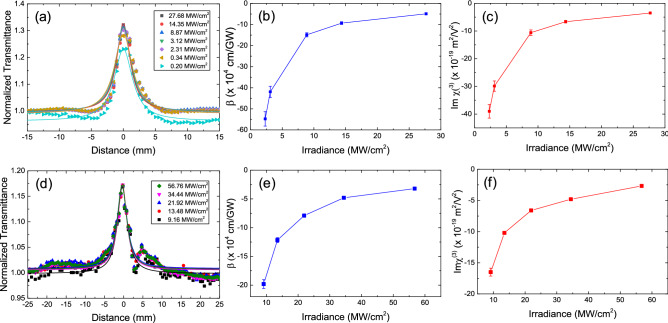
(**a**) Open aperture Z-scan curves of the α-As_2_Te_3_ film at 1,560 nm. (**b**) Nonlinear absorption coefficient (β) and (**c**) imaginary part of the third-order optical susceptibility (${\text{Im}} \chi^{(3)}$) as a function of pulse irradiance at 1,560 nm. (**d**) Open aperture Z-scan curves of the α-As_2_Te_3_ film at 1,900 nm. (**e**) Nonlinear absorption coefficient (β) and (**f**) the imaginary part of the third-order optical susceptibility (${\text{Im}} \chi^{(3)}$) as a function of pulse irradiance at 1,900 nm.

Open aperture (OA) Z-scan measurements were performed at 1,560 nm and 1,900 nm to investigate the nonlinear optical properties of the α-As_2_Te_3_, as shown in Fig. 4. In the step, a mode-locked fiber laser beam was focused through a plano-convex lens (lens 1 in Fig. 3) onto an α-As_2_Te_3_ sample mounted on a motorized translation stage. The incident laser pulses at 1,560 nm were obtained from a ~ 22.26 MHz, ~ 300 fs mode-locked fiber laser and the input laser pulses at 1,900 nm were obtained from a ~ 36.94 MHz, ~ 691 fs mode-locked fiber laser. Note that the intensity of the transmitted beam is dependent on the sample position, and it was focused using the photodetector reading of the transmittance change of the incident beam. As shown in Fig. 4a for the Z-scan curve at 1,560 nm, the normalized transmittance gradually increased as the As_2_Te_3_ sample approached the focal point (Z = 0), which was caused by the saturable absorption response. Furthermore, as the input peak intensity increased from 0.2 to 27.68 MW/cm^2^, the peaks of the OA Z-scan curves increased. These results indicate that the nonlinear optical absorption of α-As_2_Te_3_ indeed came from α-As_2_Te_3_ itself. The measured Z-scan curves were fitted with the following approximate equation^90,91^

1 $$T(z) = \sum\nolimits_{{n = 0}}^{\infty } {{{( - \beta I_{0} L_{{eff}} )^{n} {\text{ }}} \mathord{\left/ {\vphantom {{( - \beta I_{0} L_{{eff}} )^{n} {\text{ }}} {{\text{(1 + }}z^{{\text{2}}} /z_{0}^{2} {\text{)}}}}} \right. \kern-\nulldelimiterspace} {{\text{(1 + }}z^{{\text{2}}} /z_{0}^{2} {\text{)}}}}^{n} (n + 1)^{{3/2}} } \approx 1 - {{\beta I_{0} L_{{eff}} } \mathord{\left/ {\vphantom {{\beta I_{0} L_{{eff}} } {2^{{3/2}} }}} \right. \kern-\nulldelimiterspace} {2^{{3/2}} }}(1 + z^{2} /z_{0}^{2} )$$

where $T(z)$ is the normalized transmittance, $\beta$ is the nonlinear absorption coefficient, $I_{0}$ is the peak on axis-intensity at the focus, $L_{eff}$ is the effective length, $z$ is the position of the sample, and $z_{0}$ is the Rayleigh length.

Figure 4b shows the measured nonlinear absorption coefficient $\beta$ value as a function of the excitation irradiance. The nonlinear absorption coefficient values of the prepared α-As_2_Te_3_ sample vary from (− 54.8 ± 3.4)$\times$ 10^4^ cm/GW to (− 4.9 ± 0.4) × 10^4^ cm/GW as the irradiance of the input beam was increased, as shown in Fig. 4b. The experimental results showed that, the nonlinear absorption coefficient was obviously dependent on the irradiance. This phenomenon was also observed in the previous reports on topological insulators of Bi_2_Te_3_ and Bi_2_Se_3_, which also belong to group-15^97^. It was also reported in MXenes, SnSe_2_, and gold nanoprticles^63,92,93^. Figure 4d shows the measured Z-scan curves with fitting curves from the approximate equation^90,91^ at 1,900 nm. The nonlinear absorption coefficient of the sample was measured to be from (− 19.8 ± 0.8) × 10^4^ cm/GW to (− 3.2 ± 0.1) × 10^4^ cm/GW at 1,900 nm. The asymmetric shape of the measured data in Fig. 4d could be attributed to the surface non-uniformity of the deposited sample. Sample distortions, wedges, or tilting of the sample during Z-scan translation can cause unwanted fluctuations in the detected signal of the Z-scan measurement setup^98–100^. In order to avoid the unwanted fluctuations in Z-scan curve, a uniform surface of the sample is essential. A further improvement for our sample preparation process is required and will be done for the future.

It is well-known that the OA Z-scan technique is a standard method for measuring nonlinear optical responses associated with saturable absorption and two-photon absorption, even if the results could be influenced by the thermal effects caused by the input pulse laser temporal characteristics. Saturable absorption and two photon absorption are known to occur under different conditions, even if their coexistence of the two phenomena was reported^101–105^. In general, the threshold of two-photon absorption is higher than that of saturable absorption. When a high intensity beam is launched into a nonlinear optical material, saturable absorption first occurs due to the Pauli’s blocking principle^11^. As the incident beam intensity was enlarged, the carriers in the valence band absorb two photons simultaneously and the two-photon absorption process begins to appear^103–105^. However, it would be possible to induce two-photon absorption before saturable absorption occurs^101,102^. In the case of coexistence of saturable absorption and two-photon absorption, the OA Z-scan curve exhibits symmetrical dips on both sides around the center peak. Note that our measured OA Z-scan curve does not have such symmetrical dips despite of a dip on a single side. This indicates that the contribution of two-photon absorption for our measured OA Z-scan curve is not evident. Furthermore, regarding the thermal effects, which might result in a wrong interpretation on OA Z-scan measurement results, it is believed that we could rule the possibility of the intra-pulse and pulse-to-pulse cumulative thermal effects out in our measurements due to use of low pulse-energy, 703-fs pulses as an input beam even if the repletion rate is quite high (22.26 MHz)^101,106^. However, further investigations into the thermal effects needs to be conducted for the future.

It is well known that nonlinear absorption properties vary depending on the polarization status of the incident beam and the OA Z-scan measurement thus exhibits polarization-dependent curve variations^107–110^. However, we have not observed any polarization-dependent variations on the OA Z-scan curves for our prepared sample. This phenomenon could be attributed to the fact that our used As_2_Te_3_ particles are polycrystalline and bulk-structured. Note that the polarization dependence of nonlinear absorption was mostly observed in the case of crystalline or 2-D structured materials.

Table 1 summarizes the nonlinear absorption coefficients of different group-15 sesquichalcogenides and the α-As_2_Te_3_. It should be noticed that the nonlinear absorption coefficient of the α-As_2_Te_3_ is an order of magnitude larger that the values at of Bi_2_Se_3,_ Bi_2_Te_3,_ and Bi_2_TeSe_2_ at a 1.5-μm wavelength^96,97^. Since our measurements were conducted at 1.56 and 1.9 μm wavelengths, only, the comparison was limited to the measured values at 1.5-μm wavelengths. To the best of the authors’ knowledge no Z-scan measurement at a wavelength of 1.9 μm has been reported on group-15 sesquichalcogenides until now. Our Z-scan results imply that the α-As_2_Te_3_ possesses nonlinear optical response large enough for the practical implementation of as SAs.

**Table 1:** Nonlinear absorption coefficients (β) of the various TI materials and the As_2_Te_3_.

Materials	Wavelength (nm)	Nonlinear absorption coefficient (× 10^4^ cm/GW)	References
Sb_2_Te_3_	632.8	− 6.63 × 10^5^	^94^
Bi_2_Se_3_	800	− (84 ± 1.5)	^95^
Bi_2_Se_3_	532	− (2.0 ± 0.4)	^97^
Bi_2_Se_3_	800	− (6.5 ± 1.3)	^97^
Bi_2_Se_3_	1,050	− (5.5 ± 1.1)	^97^
Bi_2_Se_3_	1,550	− (2.3 ± 0.5)	^97^
Bi_2_Te_3_	1,550	− 1	^96^
Bi_2_Te_3_	532	− (3.2 ± 0.6)	^97^
Bi_2_Te_3_	800	− (2.1 ± 0.4)	^97^
Bi_2_Te_3_	1,050	− (4.7 ± 0.9)	^97^
Bi_2_Te_3_	1,550	− (3.9 ± 0.8)	^97^
Bi_2_TeSe_2_	532	− (3.4 ± 0.7)	^97^
Bi_2_TeSe_2_	800	− (5.6 ± 1.1)	^97^
Bi_2_TeSe_2_	1,050	− (8.7 ± 1.7)	^97^
Bi_2_TeSe_2_	1,550	− (5.9 ± 1.2)	^97^
Bi_2_Te_2_Se	532	− (2.6 ± 0.5)	^97^
Bi_2_Te_2_Se	800	− (3.7 ± 0.7)	^97^
Bi_2_Te_2_Se	1,050	− (9.3 ± 1.9)	^97^
Bi_2_Te_2_Se	1,550	− (7.7 ± 0.8)	^97^
α-As_2_Te_3_	1,560	(− 54.8 ± 3.4) ~ (− 4.9 ± 0.4)	This work
α-As_2_Te_3_	1,900	(− 19.8 ± 0.8) ~ (− 3.2 ± 0.1)	This work

Also, the imaginary part of the third-order optical susceptibility (${\text{Im}} \chi^{(3)}$) was calculated using the following equation^63,111^:

2 $${\text{Im}} \chi^{(3)} = \frac{{2\varepsilon_{0} c^{2} n_{0}^{2} }}{3\omega }\beta$$

where $c$ is the light speed, $\varepsilon_{0}$ is the vacuum permittivity, $\omega$ is the angular frequency, and $n_{0}$ is the refractive index. Note that the refractive index ($n_{0}$) used for this calculation was obtained from our DFT calculation results in Fig. 2c. As shown in Fig. 2c, the theoretical refractive indices of As_2_Te_3_ at a wavelength of 1.56 and 1.9 μm are 4.03 and 3.95, respectively. The imaginary parts of the third-order optical susceptibilities of As_2_Te_3_ were estimated to vary from (− 39 ± 2.4) × 10^–19^ m^2^/V^2^ to (− 3.5 ± 0.3) × 10^–19^ m^2^/V^2^ at 1,560 nm and (− 16.5 ± 0.7) × 10^–19^ m^2^/V^2^ to (− 2.8 ± 0.1) × 10^–19^ m^2^/V^2^ at 1,900 nm, respectively, depending on the irradiance of the input beam.

It was reported that the precise estimation for the imaginary part of the third-order optical susceptibility (${\text{Im}} \chi^{(3)}$) requires not only the nonlinear absorption coefficient but also the additional information of nonlinear refractive index, which can be obtained with the CA Z-scan technique^112^. However, a reasonable estimation of the third-order optical susceptibility (${\text{Im}} \chi^{(3)}$) without loss of generality, is known to be still obtainable only from OA Z-scan measurement results^63,111,113^.

### Fabrication and characterization of a saturable absorber

Two all-fiberized SAs based on α-As_2_Te_3_ were fabricated using simple sandwich structures of fiber ferrules: one is for 1.5-μm operation and the other for 1.9-μm. The prepared α-As_2_Te_3_ solution was first mixed with PVP to facilitate the formation of a film when it was dropped onto a flat surface. More precisely, 1 g of the As_2_Te_3_ bulk was grinded in a mortar to obtain α-As_2_Te_3_ powders. The α-As_2_Te_3_ particle solution was prepared using a sonication in 30 ml of distilled water without centrifugation after ultrasonication for 8 h. In order to form a composite of the As_2_Te_3_ particles and the PVP, 500 mg of PVP was mixed with the α-As_2_Te_3_ particle solution. A small amount of α-As_2_Te_3_/PVP composite solution was directly deposited onto the end surface of a FC/APC fiber ferrule and was connected to another FC/APC fiber ferrule to form a sandwich-structured SA. The insertion loss of the α-As_2_Te_3_/PVP-based SA operating at a wavelength of 1,560 nm was measured to be ~ 2.8 dB, while the value of the SA at 1,900 nm was ~ 2.5 dB.

**Figure 5:**
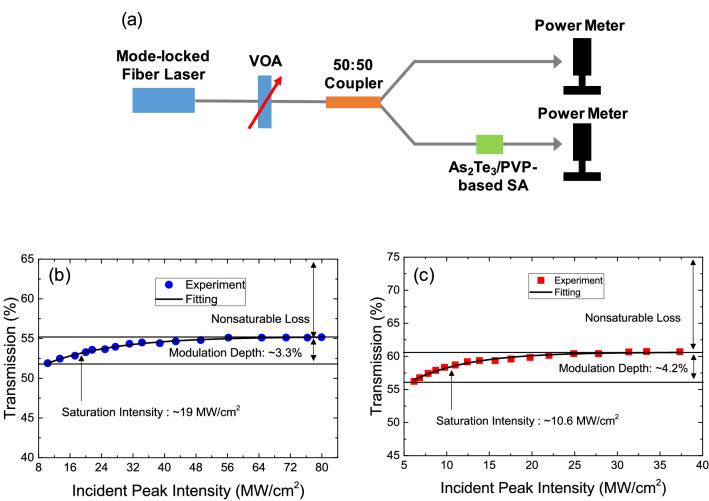
(**a**) Measured setup for nonlinear transmission of the α-As_2_Te_3_-based SA. Measured nonlinear transmission curve as a function of incident peak intensities at (**b**) 1,560 nm and (**c**) 1,900 nm.

Then, the nonlinear saturable absorption properties of both of the fabricated SAs were measured to determine their nonlinear transmission properties, as shown in Fig. 5. In these measurements, we used our custom-made mode-locked fiber lasers. The pulse width and repetition rate of our 1.56-μm mode-locked fiber laser were ~ 730 fs and ~ 22.26 MHz, respectively, whereas those of the 1.9-μm mode-locked fiber laser were ~ 703 fs and ~ 36.94 MHz, respectively. The measurement setup is shown in Fig. 5a. Figure 5b shows the measured nonlinear transmission curve of the α-As_2_Te_3_/PVP-based SA operating at 1.56 μm as a function of the incident peak power, while Figure 5c shows the curve of the SA at 1.9 μm. The modulation depths and saturation intensity were measured to be ~ 3.3% and ~ 19 MW/cm^2^ at 1.56 μm, and ~ 4.3% and ~ 10.6 MW/cm^2^ at 1.9 μm, respectively. The following formula, which is commonly used to fit the SA, was used for curve fitting^114^:

3 $$T(I) = 1 - \Delta T \cdot \exp \left(\frac{ - I}{{I_{sat} }}\right) - T_{ns}$$

where $T(I)$ is the transmission, $\Delta T$ is the modulation depth, $I$ is the input pulse energy, $I_{sat}$ is the saturation energy, and $T_{ns}$ is the nonsaturable loss.

### Implementation of pulsed fiber lasers

**Figure 6:**
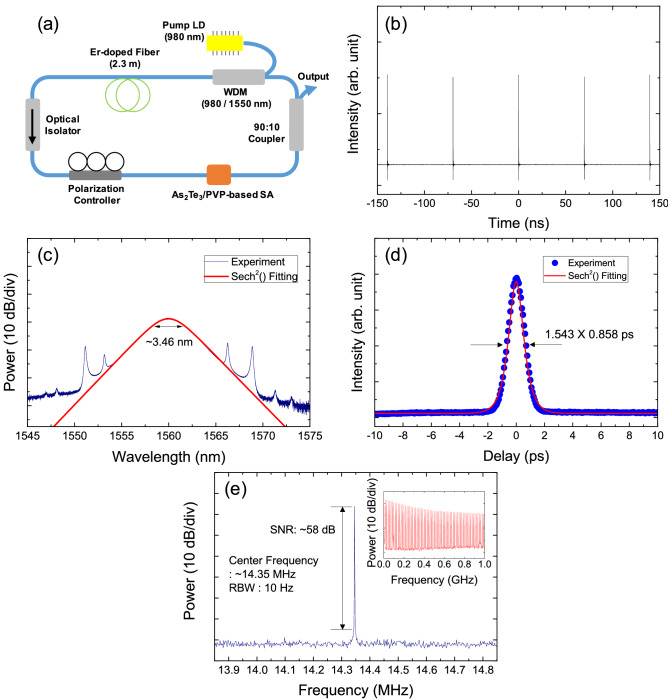
(**a**) Configuration of an erbium-doped fiber (EDF) laser cavity. Measured (**b**) oscilloscope trace, (**c**) optical spectrum, (**d**) autocorrelation trace, and (**e**) electrical spectrum of the output pulses at 1.56 μm. Inset: measured electrical spectrum over a 1-GHz span.

The experimental schematic of an Er-doped fiber laser with an α-As_2_Te_3_/PVP-based SA is shown in Fig. 6a. The α-As_2_Te_3_/PVP-based SA with an insertion loss of ~ 2.8 dB was incorporated into the cavity produce mode-locked pulses from an Er-doped fiber laser cavity. Using this fiber laser configuration, mode-locked pulses were obtainable by increasing the pump power with suitable adjustment of the polarization controller. When the pump power was above 18 mW, stable mode-locked pulses were successfully obtained in which the average output power was ~ 0.1 mW. The repetition rate and temporal period of the output mode-locked pulses were measured to be 14.35 MHz and 69.7 ns, respectively, which correspond to the fundamental resonance frequency and round-trip time of the fiberized cavity (Fig. 6b).

Figure 6c shows the optical spectrum of the output pulses together with its sech^2^ fitting curve. The center wavelength and 3-dB bandwidth were measured to be ~ 1559.9 nm and ~ 3.46 nm, respectively. Kelly sidebands were clearly observed on the optical spectrum, indicating that the mode-locked fiber laser operated in the soliton regime^115^. Next, we conducted an autocorrelation measurement by using a two-photon absorption-based autocorrelator. The temporal width of the output pulses was measured to be ~ 858 fs, as shown in Fig. 6d, and the time-bandwidth product was estimated to be 0.366, indicating that the output pulses were slightly chirped. We observed a sharp and strong peak in the electrical spectrum of the output pulses having a fundamental repetition rate of 14.35 MHz and a peak-to-background ratio of ~ 58 dB (Fig. 6e).

As shown in our theoretical investigation and linear absorption measurement, the optical absorption of α-As_2_Te_3_ was in the mid-infrared wavelength region. Therefore, to verify the applicability of the α-As_2_Te_3_/PVP-based SA to mid-infrared wavelength lasers, the prepared SA was incorporated in a Tm-Ho co-doped fiber ring cavity to generate mode-locked pulses in the 1.9 μm wavelength region. A fiber laser setup similar to Fig. 6a was used for this particular experiment. A 1-m long Tm-Ho co-doped fiber (TH512, CorActive) with 13 dB/m absorption at 1,550 nm was used as a gain medium. A 1,550-nm laser diode with a maximum pump power of ~ 297 mW was used as a pump source, which was coupled into the gain medium through a 1,550/2,000 nm wavelength division multiplexer (WDM). Stable mode-locked pulses were readily obtained when the pump power was set to be ~155 mW.

**Figure 7:**
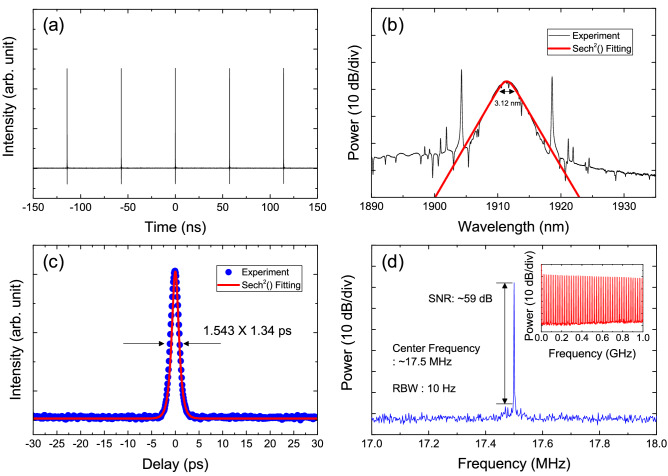
Measured (**a**) oscilloscope trace, (**b**) optical spectrum, (**c**) autocorrelation trace, and (**d**) electrical spectrum of the output pulses at 1.9 μm. Inset: measured electrical spectrum over a 1-GHz span.

Figure 7a shows the measured oscilloscope trace of the output pulses with a combination of a 16 GHz real-time oscilloscope and a 12.5-GHz photodetector. The repetition rate of the output pulses was measured to be 17.5 MHz. Figure 7b shows the measured optical spectrum of the mode-locked pulses. The center wavelength and 3-dB bandwidth were measured to be ~ 1911.4 nm and 3.12 nm, respectively. Next, an autocorrelation measurement was conducted for the output pulses. The measured temporal width of the mode-locked pulses was ~ 1.34 ps, as shown in Fig. 7c, and the time-bandwidth product was estimated to be 0.343, indicating that the output pulses were slightly chirped. Finally, the electrical spectrum was measured to check the phase noise of mode-locked pulses as shown in Fig. 7d. A strong signal peak with an electrical signal-to-noise ratio (SNR) of ~ 59 dB was clearly observed at a fundamental frequency of ~ 17.5 MHz.

We checked the long-term stability of the fabricated SAs for few days, however, we could not find any performance degradation of the SAs. We also checked the long-term stability of the mode-locked fiber lasers by monitoring the laser output for several hours but could not find any stability problem. Furthermore, we launched continuous-wave, amplified laser beams with powers of 1 W and 100 mW at 1,550 nm and 1,900 nm, respectively into our prepared As_2_Te_3_-based SAs to measure their damage thresholds. We have not observed any damage of the prepared SAs within the power levels. Therefore, it is believed that the damage threshold values of the prepared As_2_Te_3_-based SAs must be larger than 1W at 1,550 nm and 100 mW at 1,900 nm, each. However, it was impossible to measure the precise damage thresholds due to the limited availability of high power lasers in our laboratory.

## Conclusion

Here, we have conducted the first investigation of the nonlinear saturable absorption properties of α-As_2_Te_3_ using both experimental and theoretical techniques. Nonlinear absorption coefficients (β) of α-As_2_Te_3_ were measured using the OA Z-scan technique at a wavelength of 1,560 nm and 1,900 nm. They were found to vary in a range from (− 54.8 ± 3.4)$\times$ 10^4^ cm/GW to (− 4.9 ± 0.4)$\times$ 10^4^ cm/GW at 1,560 nm and from (− 19.8 ± 0.8)$\times$ 10^4^ cm/GW to (− 3.2 ± 0.1)$\times$ 10^4^ cm/GW at 1,900 nm, respectively. Interestingly, the nonlinear absorption coefficient of α-As_2_Te_3_ at 1.56 μm was found to be an order of magnitude larger than the previously reported values of other group-15 sesquichalcogenides such as Bi_2_Se_3,_ Bi_2_Te_3,_ and Bi_2_TeSe_2_. Also, the estimated imaginary parts of the third-order optical susceptibilities (${\text{Im}} \chi^{(3)}$) of the prepared As_2_Te_3_-sample vary from (− 39 ± 2.4)$\times$ 10^–19^ m^2^/V^2^ to (− 3.5 ± 0.3)$\times$ 10^–19^ m^2^/V^2^ at 1,560 nm and (− 16.5 ± 0.7)$\times$ 10^–19^ m^2^/V^2^ to (− 2.7 ± 0.1)$\times$ 10^–19^ m^2^/V^2^ at 1,900 nm, respectively. Furthermore, it was also shown that α-As_2_Te_3_ can serve as a base material for near-infrared SAs to generate ultrafast mode-locked pulses that can cover a bandwidth of 1.5–1.9 μm. The electric band structure analysis showed that the operating bandwidth was expandable to ~ 3.7 μm, indicating that it can cover a wide range of wavelength, including the mid-infrared region.

Arsenic telluride is known to be a good thermoelectric material but is still and unknown material in the field of optics and photonics. We believe that our work reveals the significant potential of α-As_2_Te_3_ in the field of optics and photonics. Furthermore, our work is believed to provide a meaningful database for nonlinear optical materials in the fields of lasers and photonics and suggest a new, nonlinear saturable absorption material for the implementation of ultrafast lasers.

## Methods

### Analysis

The elemental composition of a prepared sample was analyzed using an EDS measurement (VEGA3, TESCAM). Raman spectroscopy measurement was performed using a LabRam Aramis (Horiba Jovin Yvon) at room temperature under excitation by 532 nm laser with output power of ~ 0.5 mW. XPS measurements were carried out on a K-alpha (Thermo Scientific Inc., UK) using an Al Ka μ-focused monochromator (1,486.6 eV) with a 400 μm spot size and energy step size of 0.1 eV. The FWHM of energy resolution is 0.7 eV measured in the Ag 3d^5/2^ peaks, and an 180° double focusing hemispherical analyzer with a 128-channel detector was employed. The energy range was from 100 to 4,000 eV, and the base pressure was 2.9 $\times$ 10^–9^ mbar.

### Density functional theory calculations

We performed the DFT calculation with spin–orbit coupling by employing gradient-density approximation and Perdew–Burke–Ernzerh of exchange–correlation parameterization as implemented in the Atomistic Toolkit package^116,117^. The density mesh cutoff was 150 hartree and the 2 $\times$ 7 $\times$ 3 Monkhorst–Pack grid was used for k-space sampling to calculate the electronic band structure and dielectric constant of α-As_2_Te_3_. To obtain optical absorption spectrum, the susceptibility tensor was calculated using the Kubo–Greenwood formula shown below^118^:

4 $$\chi_{i,j} (\omega ) = - \frac{{e^{2} \hbar^{4} }}{{m^{2} \varepsilon_{0} V\omega^{2} }}\sum\limits_{n,m} {\frac{{f(E_{m} ) - f(E_{n} )}}{{E_{nm} - \hbar \omega - i\hbar \Gamma }}\pi_{nm}^{i} \pi_{nm}^{j} }$$

where *f* is the Fermi distribution function, *Γ* is the broadening parameter,$\pi_{nm}^{i}$ is the *i*-component of the dipole matrix element between states *n* and *m*, and *V* is the volume of the considered system. The frequency-dependent dielectric constant was estimated using the relation of $\varepsilon (\omega ) = 1 + \chi (\omega )$ and the optical absorption spectrum was finally obtained from the imaginary part of the dielectric constants.

### Linear absorption measurement

The linear absorption of the α-As_2_Te_3_/PVP composite was measured by using a spectrophotometer (UV-3600PLUS, Shimadzu) from 1000 to 3300 nm.

### Z-scan measurement

As the input light source, homemade mode-locked fiber lasers operating at 1.56 and 1.9 μm were used. The beam splitter was used to split the input beam into two parts. One of the two parts was directed to a power meter to monitor the reference beam (Power meter 1), while the other was focused through a plan-convex lens and is was vertically directed to the α-As_2_Te_3_ sample. The α-As_2_Te_3_ sample was placed on the translation stage and moved gradually across in the propagation direction. The transmittance through the sample was measured using a power meter (Power meter 2)^53^.

### Nonlinear saturable absorption measurement

Homemade mode-locked fiber lasers (center wavelength = 1.56 μm, repetition rate = 22.26 MHz, and temporal width = 730 fs/center wavelength = 1.9 μm, repetition rate = 36.94 MHz, and temporal width = 703 fs) are used to measure the nonlinear transmission curve of the prepared SA. A fiber-based variable optical attenuator was used to adjust the optical power of the input mode-locked pulses. An input mode-locked pulses were divided into two ports using a 3-dB coupler. One of the two ports was connected to the prepared α-As_2_Te_3_/PVP-based SA, while the other was directly connected to a power meter to monitor the input power of the SA. Another power meter was used to measure the output power from the α-As_2_Te_3_/PVP-based SA for comparison with the input power^48,53^.

### Fabrication of α-As_2_Te_3_-based saturable absorber

Bulk As_2_Te_3_ crystals were grinded in a mortar to obtain α-As_2_Te_3_ powders. The α-As_2_Te_3_ particle solution was prepared using a sonication in a distilled water without centrifugation after ultrasonication for 8 h. Then, the PVP was mixed into the α-As_2_Te_3_ particle solution. A small amount of α-As_2_Te_3_/PVP composite solution was dropped on an FC/APC fiber ferrule and then dried at room temperature for 8 h^119^.
